# China's regulatory regime for medical biotechnology: evolution, challenges, and future perspectives

**DOI:** 10.3389/fmedt.2026.1805452

**Published:** 2026-06-09

**Authors:** Enkai Zhang

**Affiliations:** School of Life Sciences, Zhejiang Chinese Medical University, Hangzhou, China

**Keywords:** biorisk, biosafety, biosecurity, China, dual-use research, medical biotechnology, regulatory regime

## Abstract

Modern medical biotechnology, while reshaping human healthcare and enhancing social welfare, may also pose biological risks. During the past 40 years, China has made considerable strides in becoming a global leader in biotechnology, necessitating a systematic review of the evolution, current state, and challenges of its regulatory framework. This paper traces the policy trajectory and institutional evolution of China's medical biotech regulation and conducts a policy evaluation within the historical context based on a comprehensive review of laws, regulations, and ministerial rules issued since the 1980s. The biorisk management system is divided into three components and China's policy evolving process into three stages. The analysis is focused on the driving forces of the regulatory policies, the shift of regulatory philosophy, the expansion of regulatory coverage, the current legal and organizational frameworks, and the challenges faced. We find that China's medical biotech regulatory policies have evolved over the past 40 years into a comprehensive regime, spanning the full cycle of research, application and export and covering the dual dimension of security and ethics. It is now supported by multi-tiered laws and regulations, and executed by specialized coordination mechanisms. However, the existing system still falls short in addressing the risks of technological convergence, clarifying the regulatory boundary, strengthening ethical constraints, and enhancing public engagement. Accordingly, we recommend that the agility, clarity, and inclusiveness of China's medical biotech governance should be further improved.

## 1 Introduction

Modern biotechnology begins with the understanding of the structure, replication and translation of DNA and involves the application of *in vitro* nucleic acid techniques, including recombinant DNA and direct injection of nucleic acid into cells or organelles, or fusion of cells beyond the taxonomic family (1, 2). The rapid progress of modern biotechnology, while enhancing human health and social welfare, can adversely affect the health of humans, animals, plants and agriculture, and the environment through accidental release, and inadvertent or deliberate abuse and misuse of pathogenic agents and related resources. Such biological risks and threats have aroused safety and security concerns worldwide. The U.S. was the first to take regulatory actions. The National Institutes of Health published a set of research guidelines in 1976 and the Reagan Administration created a Coordinated Framework for Regulation of Biotechnology in 1986 (3). Built mainly on the U.S. experience, the World Health Organization (WHO) developed a global guidance framework for the governance of biorisks in 2022 (4).

Generally, a safe, secure and responsible biorisk management system is composed of three parts: biosafety, biosecurity and oversight of dual-use research. Biosafety refers to principles, technologies, practices, and measures implemented to prevent the risk of *unintentional* exposure to, contamination with, release of, or harm from pathogens, toxins, and biological materials. Biosecurity refers to the protection, control, and accountability measures implemented to prevent the loss, theft, misuse, diversion, unauthorized possession or transfer, or *intentional* release of pathogens, toxins, biological materials, and related information, expertise, equipment, technology and intellectual property (4–6). Thus, biosafety is usually limited to the laboratory environment and involves the protection of humans, animals, and environmental resources from the biological assets (e.g., harmful bacteria and viruses); while biosecurity can be applied both inside and outside of laboratories and its end goal is to protect the biological assets themselves from threats imposed upon them (7, 8).

Dual-use research is the research conducted for peaceful and beneficial purposes that has the potential to produce knowledge, information, methods, products or technologies that could also be *intentionally* misused to endanger the health of humans, plants and agriculture, and the environment (4). A biorisk management framework for dual-use research relies on three core pillars: research excellence, bioethics, and laboratory biosafety and security (8). As dual-use research can be associated with both unintentional accidents and deliberate misuse, it overlaps with laboratory biosafety and biosecurity (9).

The applications of modern biotechnology have traversed through all the scientific disciplines in agriculture, industry, and most significantly, medical science. Modern medical biotechnology, built on decades of breakthroughs from molecular biology and synthetic biology to genetic engineering, is reshaping human healthcare by developing innovative diagnostic modalities, targeted therapies, and preventive measures (10). Meanwhile, its misuse and dual-use problem has come to the center of ethical debates.

In China, the concept of “biological security” (*shengwu anquan*) has developed into a broad meaning, covering the management of the following biorisks: (1) major emerging and sudden-onset infectious diseases, as well as animal and plant epidemics; (2) biotech research, development, and application; (3) pathogenic microbe laboratories; (4) human genetic resources and biological resources; (5) invasion of alien species and protecting biodiversity; (6) biological terrorism; and (7) biological weapon threats. Its medical biotech regulatory regime mainly comprises the laboratory biosafety and biosecurity supervision, dual-use research oversight, and import and export control of dual-use items. This paper focuses on this regime and conducts a systematic review based on the analytical framework of “historical evolution → current system → core challenges → improvement path”. Section 2 traces the policy trajectory over the past 40 years. National laws and policies regulating healthcare, biological and medical technologies, microbiological and biomedical laboratories, and dual-use items are collected from the National Laws and Regulations Database and the PKULAW Database with the time scope from 1985 to 2025. Section 3 provides a deep insight into the current system from two perspectives: legal framework and organizational structure. Section 4 analyzes the main challenges faced. Section 5 makes suggestions for further improvement in the future. Section 6 concludes.

## 2 Policy evolution

China's regulatory system for modern medical biotechnology started in the 1980s and has undergone three stages. This process not only reflects the profound impact of international experience and major public health events, but also embodies the continuous driving force of ethical and security challenges brought by technological breakthroughs on institutional construction.

### 2.1 Initial exploration

The last two decades of the 20th century saw the initial exploration of China's modern medical biotech regulatory system. Basic regulations for risk classification of pathogenic microorganisms, management of experimental animals, and prevention and control of infectious diseases were initially introduced. At the same time, with the rise of genetic engineering technology and the deepening of international medical research cooperation, regulatory measures for the safety management of genetic engineering and human genetic resources began to emerge. However, policies in this stage were highly decentralized.

#### 2.1.1 Biosafety and biosecurity

China's biorisk management policy began with the laboratory biosafety and biosecurity supervision in the mid-1980s (Table 1). Based on the *Regulations on the Management of Microbial Strain Preservation*, which was provisionally implemented in 1979 and formally enforced in 1986, the Ministry of Health issued the *Measures for the Preservation of Medical Microbial Strains* in 1985. The first of its kind, the policy drew upon and incorporated the risk group classification of pathogenic microorganisms by the WHO *Laboratory Biosafety Manual* and the U.S. *Biosafety in Microbiological and Biomedical Laboratories* (BMBL). It classified laboratories into four categories based on the risk of bacterial strains. The goal was to help biomedical laboratories tailor their safety and security measures to the level of biological hazards and promote safer handling and storage of potentially hazardous microorganisms. Two years later, the first BSL-3 laboratory was established in China (11). In 1989, the Ministry of Health formulated the *Implementation Rules for the Management of Medical Laboratory Animals* in accordance with the *Regulations on the Management of Experimental Animals* enforced in the previous year. Also the first of its kind, it stipulated the establishment of management institutions at different governmental levels and made clear requirements on testing standards for medical experimental animals, as well as the quarantine, conservation, introduction, supply, and application of such animals. Besides, the *Law on the Prevention and Treatment of Infectious Diseases* and its implementing regulation were enacted respectively in 1989 and 1991. Both listed and classified 29 infectious bacteria or viruses, and set strict requirements for the epidemic prevention institutions and the pathogenic microorganism experimental institutions on the storage and transportation of the listed bacteria and viruses. To align with the law, the revision of the *Criminal Law* in 1997 imposed penalties on anyone who committed the crime of spreading infectious disease strains or viruses.

**Table 1:** China's medical biotech regulatory policies in the 1980s and the 1990s.

Legal level	Year	Title of laws, regulations, and ministerial rules	Issuing body
Law	1989	Law on the Prevention and Treatment of Infectious Diseases (Law on PTID)	NPC
	1997	Criminal Law (1997 Revision)	NPC
Regulation	1986	Regulations on the Management of Microbial Strain Preservation	NSTC
	1988	Regulations on the Management of Experimental Animals	NSTC
	1991	Regulations on the Prevention and Treatment of Infectious Diseases	MH
Ministerial rule	1985	Measures for the Preservation of Medical Microbial Strains (Repealed 2010)	MH
	1989	Implementation Rules for the Management of Medical Laboratory Animals (Repealed 2002)	MH
	1993	Measures for the Safety Management of Genetic Engineering (Repealed 2025)	NSTC
	1993	Key Points for the Quality Control in Clinical Research of Human Somatic Cell Therapy and Gene Therapy	MH
	1998	Interim Measures for the Administration of Human Genetic Resources	MST and MH

NPC, National People's Congress; NSTC, National Science & Technology Committee; MST, Ministry of Science & Technology; MH, Ministry of Health. MST replaced NSTC in 1998.

#### 2.1.2 Oversight of dual-use research

Regulatory policies for dual-use research began to emerge in the early 1990s (Table 1). The first was the *Measures for the Safety Management of Genetic Engineering* enforced in 1993. The policy classified genetic-engineering-related activities into four safety levels based on the potential degree of risks, and established a centralized approval system. Experimental research, intermediate testing and industrial production were all under supervision, as well as the release of genetic engineering bodies and the use of genetic engineering products. In the same year, the Ministry of Health issued the *Key Points for Quality Control in Clinical Research of Human Somatic Cell Therapy and Gene Therapy*. The document was based on the *Points to Consider in Human Somatic Cell Therapy and Gene Therapy* released in 1991 by the U.S. FDA Center for Biologics Evaluation and Research. It defined gene therapy as a medical intervention based on modification of genetic materials of human living cells and extended the regulatory scope of genetic engineering technology to clinical research. The second was the *Interim Measures for the Administration of Human Genetic Resources* co-formulated by the Ministry of Science & Technology and the Ministry of Health in 1998. The policy was promoted by and resulted from widespread concerns over research ethics and human genetic resource conservation arising from the U.S.-China gene collection and research cooperation projects conducted between 1995 and 1997 in China (12, 13). Thus, it covered a broad area from collection, research, and development to trading and exporting of human genes in China.

### 2.2 Rapid development

Driven by multiple factors such as international biosecurity incidents, the domestic Severe Acute Respiratory Syndrome (SARS) epidemic, and ethical controversies surrounding emerging technologies, China's medical biotech regulatory policies developed rapidly during the first two decades of the 21st century. The regulatory scope expanded and the regulatory philosophy gradually shifted, laying an important foundation for the integration of the subsequent legal system.

#### 2.2.1 Biosafety and biosecurity

The 9/11 attacks and the anthrax incidents in the U.S. in 2001 aroused worldwide concern over biosafety and biosecurity and pushed countries to strengthen control measures for biological agents and toxins. In China, the Ministry of Foreign Trade & Economic Cooperation (currently the Ministry of Commerce) issued an *Urgent Notice on Preventing Biological and Chemical Weapons such as Anthrax from Entering China through International Express Channels* in November 2001. The following year, the *Regulations on the Export Control of Dual-use Biological Agents and Related Equipment and Technologies* was promulgated. It established a prevention mechanism against the risk of misuse of dual-use biological agents, equipment and technologies. This marked the extension of China's biosafety and biosecurity regulation from preventing domestic risks to international risks. Dual-use biological agents were defined as pathogens, toxins, and genetic materials that could be used for peaceful purposes such as medical treatment, prevention, protection, and defense, as well as for non-peaceful purposes such as the development and production of biological weapons. To implement the export regulation, administrative measures for export registration and licensing of dual-use items and technologies, as well as relevant export control lists, were issued between 2002 and 2006 (Table 2).

**Table 2:** China's medical biotech regulatory policies in the 2000s and the 2010s.

Legal level	Year	Title of laws, regulations, and ministerial rules	Issuing body
Law	2015	Counterterrorism Law	NPC
	2019	Vaccine Administration Law	NPC
	2019	Basic Healthcare and Health Promotion Law (BHHP Law)	NPC
	2019	Medicinal Product Administration Law (MPA Law) (2019 Revision)	NPC
Regulation	2002	Regulations on the Export Control of Dual-use Biological Agents and Related Equipment and Technologies	SC
	2003	Regulations on the Responses to Public Health Emergencies	SC
	2004	Regulations on the Biosafety Management of Pathogenic Microbe Laboratories (Regulations on BMPML)	SC
	2019	Regulations on the Administration of Human Genetic Resources (Regulations on AHGR)	SC
Ministerial rule	2002	Measures for the Administration of the Export Registration of Sensitive Items and Technologies	MFTEC
	2002	General Guidelines for the Biosafety in Microbiological and Biomedical Laboratories	MH
	2003	Ethical Principles for Guiding Human Embryonic Stem Cell Research (Ethical Principles for GHESCR)	MST and MH
	2005	Measures for the Examination and Approval of the Biosafety Management of Highly Pathogenic Microbe Laboratories for Animal Pathogens	MA
	2005	Provisions on the Transport Management of Highly Pathogenic Bacterial (Viral) Strains or Samples that Can Infect Humans	MH
	2005	List of Animal Pathogenic Microbes	MA
	2005	Measures for the Administration on Import and Export License for Dual-use Items and Technologies (Measures for AIEL)	MC and GAC
	2006	List of Pathogenic Microbes Transmitting among Humans	MH
	2006	Measures for the Administration of the Biosafety Approval of Laboratories and Experimental Activities Related to Highly Pathogenic Microbes Transmitting among Humans	MH
	2006	Measures for the Biosafety Environmental Management of Pathogenic Microbe Laboratories (Measures for BEMPML)	GAEP
	2006	Export Control List of Dual-use Biological Products and Affiliated Equipment and Technologies	MC
	2009	Measures for the Administration of Institutions Storing Pathogenic Bacterial (Viral) Strains Transmitting among Humans (Measures for AISPBSTH)	MH
	2009	Measures for the Management of the Clinical Application of Medical Technologies	MH
	2011	Measures for the Review of the Construction of High-level Pathogenic Microbe Laboratories	MST
	2015	Interim Measures for the Management of Stem Cell Clinical Research	NHFPC
	2016	Measures for the Ethical Review of Biomedical Research Involving Humans	NHFPC
	2017	General Guidelines for Biosafety in Pathogenic Microbial Laboratories	NHFPC
	2017	Measures for the Safety Management of Biotech Research and Development (Measures for SMBRD)	MST
	2018	Measures for the Management of the Clinical Application of Medical Technologies (Measures for MCAMT)	NHC

NPC, National People's Congress; SC, State Council; MFTEC, Ministry of Foreign Trade &Economic Cooperation; MH, Ministry of Health; MST, Ministry of Science & Technology; MA, Ministry of Agriculture; MC, Ministry of Commerce; GAC, General Administration of Customs; GAEP, General Administration of Environmental Protection; MEP, Ministry of Environmental Protection; NHFPC, National Health & Family Planning Commission; NHC, National Health Commission.

MC replaced MFTEC in 2003. MEP replaced GAEP in 2008. NHFPC replaced MH in 2013 and was replaced by NHC in 2018.

The outbreak of the SARS pandemic, and the related laboratory infection and pathogen leakage during 2002 and 2003 had two profound impacts on China's biorisk management: one was the reform of the monitoring, reporting, and response system for infectious diseases; the other was the enhancement of laboratory biosafety and biosecurity oversight system. In 2004, the *Law on the Prevention and Treatment of Infectious Diseases* was amended to establish a four-dimensional infectious disease prevention and control system, including prevention and early warning, epidemic control, medical treatment, and supervision and protection. This amendment significantly changed the previous legal emphasis on *ex post* control and shifted towards a system that emphasized both prevention and control. Based on the related provisions of the Law, the laboratory biosafety and biosecurity oversight system was upgraded to a comprehensive one, ensuring that every aspect was under surveillance. Notably, the *Regulations on the Biosafety Management of Pathogenic Microbe Laboratories* (*Regulations on BMPML*) was implemented in 2004. It divided pathogenic microorganisms into four risk groups in a descending order the same way as the *Measures for the Preservation of Medical Microbial Strains* published in 1985 (see Section 2.1), but definitely categorized groups one and two as highly pathogenic. In accordance with that regulation and in response to global infectious diseases and pandemics like H1N1 influenza, avian influenza, and Middle East Respiratory Syndrome (MERS), a series of ministerial measures went into effect during the following 15 years. They covered every link of laboratory biosafety and biosecurity oversight from laboratory construction and experimental activities to bacterial and toxin use, transportation, and storage, as well as waste disposal. Specifically, the lists of animal pathogenic microbes and pathogenic microbes transmitting among humans were released for the first time based on the risk grouping by the *Regulations on BMPML*.

More importantly, three laws were enacted during this period to manage biorisks based on different legislative considerations. The *Counterterrorism Law* promulgated in 2015 stipulated strict supervision and control of infectious disease pathogens and related substances to prevent their flow into illegal channels and required immediate control and report in case of theft, robbery and loss. The *Vaccine Administration Law* of 2019 mainly focused on biosafety and biosecurity oversight throughout the entire process from vaccine research and development to production, distribution and immunization, particularly the strict management of pathogenic microbes (such as bacterial strains) used for vaccines. The *Basic Healthcare and Health Promotion Law* of 2019, from the perspective of enhancing the basic healthcare system, reiterated the establishment of a prevention, control and blocking system against infectious diseases.

#### 2.2.2 Oversight of dual-use research

With respect to the dual-use research, a series of laws and regulations were enforced during this period in response to technological breakthroughs in cell therapy and gene editing (Table 2).

The discovery of human embryonic stem cells by U.S. biologists in 1998 (14), while opening new doors for disease modeling, drug screening, and cell therapy, also sparked intense ethical debates worldwide about the origin of life, the moral status of human embryos, and the research oversight (15, 16). In China, the *Ethical Principles for Guiding Human Embryonic Stem Cell Research* was issued in 2003 as the first ethical norm in this field. It complied with internationally recognized ethical guidelines on life sciences, and clarified the fundamental stance of “supporting therapeutic research while opposing reproductive research”. However, the rapid development of cell therapy raised medical quality and safety concerns due to the misuse and abuse of such technologies in clinical application. Therefore, the *Measures for the Management of the Clinical Application of Medical Technologies* were released in 2009 (*2009 Measures*). It proposed for the first time at the national level the classification and grading management of medical biotechnologies by dividing them into three categories. Technologies involving significant ethical issues and high biorisks, requiring further verification of safety and effectiveness, or using scarce resources were classified as the third category under the strictest control. Nineteen technologies were listed, including cloning therapy, autologous stem cell and immune cell therapy, gene therapy, allogeneic stem cell transplantation, and homologous organ transplantation.

However, the *2009 Measures* applied only to the admission of clinical applications of medical biotechnology and did not cover clinical research. Emerging medical biotechnologies were in a gray area. Many medical institutions, driven by financial interests, pushed stem cell therapies still in the research phase directly into clinical practice to cheat patients and make illegitimate benefits. To curb the chaos, the Chinese government suspended all unapproved stem cell treatments in 2012 and promised to develop detailed management measures. With the reform of the government administrative approval system in 2015, the National Health and Family Planning Commission, which had replaced the Ministry of Health two years earlier, repealed the *2009 Measures*, canceled the approval requirement for clinical applications of the third-category medical biotechnology, and tried to establish a “negative list” system. In the same year, the *Interim Measures for the Management of Stem Cell Clinical Research* were issued and made definite distinction between clinical research and application of new medical biotechnologies. Three years later, the new *Measures for the Management of the Clinical Application of Medical Technologies* (*2018 Measures*) formally established the negative list system which classified forbidden and restricted technologies. However, the policy explicitly excluded its applicability to the cell therapy technology, including stem cell and immune cell therapy, gene therapy, etc.

Meanwhile, the development of CRISPR/Cas9 gene editing technology in 2012 revolutionized molecular biology and held profound potential for gene and tumor therapy, gene screening and detection, and the breeding and genetic modification of animals and plants (17). In 2015, a group of Chinese scientists used the technology for the first time to modify a pathogenic gene in human embryos (18). The breakthrough, while bringing scientific possibilities, sparked extensive debates on ethics, safety, and social impact. It directly prompted the revision and upgrading of relevant regulatory rules in China. In the following two years, the *Measures for the Ethical Review of Biomedical Research Involving Humans* and the *Measures for the Safety Management of Biotechnology Research and Development* were released. The former provided that medical and health institutions engaged in biomedical research involving humans should establish ethics committees and take effective measures to ensure their independent ethical reviews. The latter divided the biotech R&D activities into three categories according to their potential risks, and those involving human gene editing and highly pathogenic microbes, among others, were defined as the highest risk.

In 2018, a team of Chinese scientists used CRISPR/Cas9 technology to conduct gene editing on the embryos of twin baby girls so that they could naturally resist AIDS after birth. This incident, due to its enormous ethical, safety, and social risks, quickly sparked strong condemnation and deep reflection on a global scale (19). In China, the incident reflected the poor awareness of ethical norms, the soft legal constraints on researchers, and the weak effectiveness of the existing regulatory system on emerging medical biotechnologies. In response, the *Basic Healthcare and Health Promotion Law* and the second revision of the *Medicinal Product Administration Law* (*MPA Law*) were implemented in 2019. Both incorporated for the first time in the law ethical review provisions for medical researches and clinical trials of drugs and medical devices. In addition, a series of laws, government regulations and ministerial measures were drafted for public comments in 2019, such as the *Draft Biosecurity Law*, the *Proposed Regulations on the Clinical Application of Biomedical New Technologies*, the *Proposed Regulations on the Safety Management of Biotechnology Research and Development*, and the *Proposed Interim Measures for the Management of Clinical Research and Translational Application of Somatic Cell Therapies*.

### 2.3 Maturity

While the regulatory reflection on human embryonic gene editing and CRISPR-edited babies was still in progress, COVID-19 broke out in China at the turn of the third decade of the 21st century. In the following three years, the disease evolved into a global pandemic of the widest impact in the past century (20).

As discussed above, before the crisis, China's biorisk regulatory measures were scattered in various laws, regulations, ministerial rules, administrative documents, and policy guidelines. The pandemic completely changed the regulatory philosophy from a decentralized and passive response to a full chain and active risk prevention and control. Such an approach needed an overarching law. Therefore, the *Biosecurity Law* was adopted through an expedite legislative process in 2020, only one year after it was introduced to the national legislative organ. The law defined the concept of “biosecurity” in a broad sense as “a state in which a country can effectively prevent and respond to the threat of hazardous biological agents and related factors; its biotechnology can develop steadily and healthily; neither the lives and health of its people nor its ecosystem is relatively in danger or under threat; and the country is equipped with such capabilities in the biological field to maintain national security and sustainable development”. It covered the management of seven categories of biorisks, including epidemics, biotech research and application, laboratory safety and security, genetic resources, alien species, bioterrorism, and bioweapons. To align with the *Biosecurity Law*, related laws, regulations and ministerial rules were promulgated or revised over the following years (Table 3).

**Table 3:** China's medical biotech regulatory policies in the 2020s.

Legal level	Year	Title of laws, regulations, and ministerial rules	Issuing body
Law	2020	Civil Code	NPC
	2020	Export Control Law (EC Law)	NPC
	2020	Biosecurity Law	NPC
	2020	Amendment (XI) to the Criminal Law	NPC
	2021	Law on the Advancement of Science and Technology (Law on AST) (2021 Revision)	NPC
	2021	Law on Doctors	NPC
	2025	Law on the Public Health Emergency Response	NPC
Regulation	2024	Regulations on the Export Control of Dual-use Items (Regulations on ECDUI)	SC
	2024	Export Control List of Dual-use Items (EC List of DUI)	SC
	2025	Regulations on the Administration of Clinical Research on and Clinical Translational Application of New Medical Biotechnologies (Regulations on ACRCTANMB)	SC
Ministerial rule	2023	Interim Measures for Scientific and Technological Ethical Review (Measures for STER)	MST, NHC, ME, MARA, etc.
	2023	Measures for the Ethical Review of Life Science and Medical Research Involving Humans (Measures for ERLSMRH)	MST, ME, NHC, etc.
	2023	List of Pathogenic Microbes Transmitting among Humans (List of PMTH) (2023 revision)	NHC
	2023	Norms and Guidelines for the Approval of Experimental Activities Involving Highly Pathogenic or Suspected Highly Pathogenic Microbes and for the Transportation Licensing and Approval of Highly Pathogenic Microbes	NHC
	2023	Rules for the Implementation of the Regulations on the Administration of Human Genetic Resources (Rules for the Implementation of the Regulations on AHGR)	MST
	2024	Ethical Guidelines for Human Non-Human Animal Chimera Research (Ethical Guidelines for HNHACR)	NSTEC
	2024	Ethical Guidelines for Human Genome Editing Research (Ethical Guidelines for HGER)	NSTEC
	2024	Ethical Guidelines for Brain Computer Interface Research (Ethical Guidelines for BCIR)	NSTEC
	2025	Ethical Guidelines for Human Derived Organoids Research (Ethical Guidelines for HDOR)	NSTEC
	2025	Measures for the Administration of the Biosafety Approval of Experimental Activities Related to Highly Pathogenic Microbes Transmitting among Humans (Measures for ABAEA)	NHC

NPC, National People's Congress; SC, State Council; MST, Ministry of Science & Technology; NHC, National Health Commission; ME, Ministry of Education; MARA, Ministry of Agriculture & Rural Affairs; NSTEC, National Science & Technology Ethics Committee. MARA replaced MA (Ministry of Agriculture) in 2018.

#### 2.3.1 Biosafety and biosecurity

For the management of laboratory biosafety and biosecurity, the *List of Pathogenic Microbes Transmitting among Humans* was updated in 2023. In the same year, the *Norms and Guidelines for the Approval of Experimental Activities Involving Highly Pathogenic or Suspected Highly Pathogenic Microbes and for the Transportation Licensing and Approval of Highly Pathogenic Microbes* were issued. In 2025, the *Measures for the Administration of the Biosafety Approval of Experimental Activities Related to Highly Pathogenic Microbes Transmitting among Humans* replaced its previous version issued in 2006.

For the prevention of international biorisks, China established an integrated and unified export control system through enacting the *Export Control Law* in 2020 and issuing the *Regulations on the Export Control of Dual-use Items* in 2024. The latter replaced the *Regulations on the Export Control of Dual-use Biological Agents and Related Equipment and Technologies* (see Section 2.2) and three other export control regulations on nuclear, missile, and chemical products and technologies implemented in the late 1990s and the early 2000s. The “dual-use items” were defined as “goods, technologies, and services that are for both civil and military purposes or contribute to an increase in military potential, especially those that can be applied to designing, developing, producing, or using weapons of mass destruction and their means of delivery, including relevant technical information and other data”. These items fell into ten categories. Microorganisms and toxins were among the first category of the export control list, including pathogenic microbes and toxins that can be transmitted among humans and between humans and animals, as well as genetic materials and genetically modified organisms, and plant pathogenic microbes. They were listed under the classification numbers of 1C351, 1C353, and 1C354 respectively, and their related technologies and equipment were under the classification numbers of 1E301, 2B352, and 2E301 respectively.

#### 2.3.2 Oversight of dual-use research

The oversight of dual-use research was another focal issue of this stage and the construction of the ethical review system was the key. In 2020, the National Science & Technology Ethics Committee was established to strengthen the overall planning, standardization, guidance, and coordination of the ethical review system for science and technology. This was incorporated into the revision of the *Law on the Advancement of Science and Technology* in 2021. The committee established three subcommittees: life sciences, medical science, and artificial intelligence (AI). In 2023, the *Measures for Ethical Review of Life Science and Medical Research Involving Humans* (*2023 Measures for ERLSMRH*) was issued, which clarified the scope of ethical review, emphasized the independence of the review board, and standardized the review procedure. Thereafter the Life Sciences Ethics Subcommittee and the Medical Ethics Subcommittee released three ethical guidelines on the researches involving human non-human animal chimera, human genome editing, and human derived organoids.

Moreover, to strengthen the punishment for the violation of the *Biosecurity Law*, the *Amendment (XI) to the Criminal Law* introduced three new crimes: illegal collection of human genetic resources and smuggling of human genetic resource materials; illegal implantation of gene-edited or cloned embryos; and illegal introduction, release, or abandonment of invasive alien species. Besides, the *Law on Doctors* issued in 2021 provided that any doctor who seriously violated medical ethical norms should be banned from engaging in medical and health services or clinical research for five years or even life.

## 3 Current oversight system

After 40 years of gradual development, an integrated regulatory regime for medical biotechnology has finally taken shape in China in the early 2020s. It brings decentralized oversight measures into a single integrated framework and strives to achieve a dynamic balance among innovation, security, and ethics.

### 3.1 Legal framework

**Figure 1:**
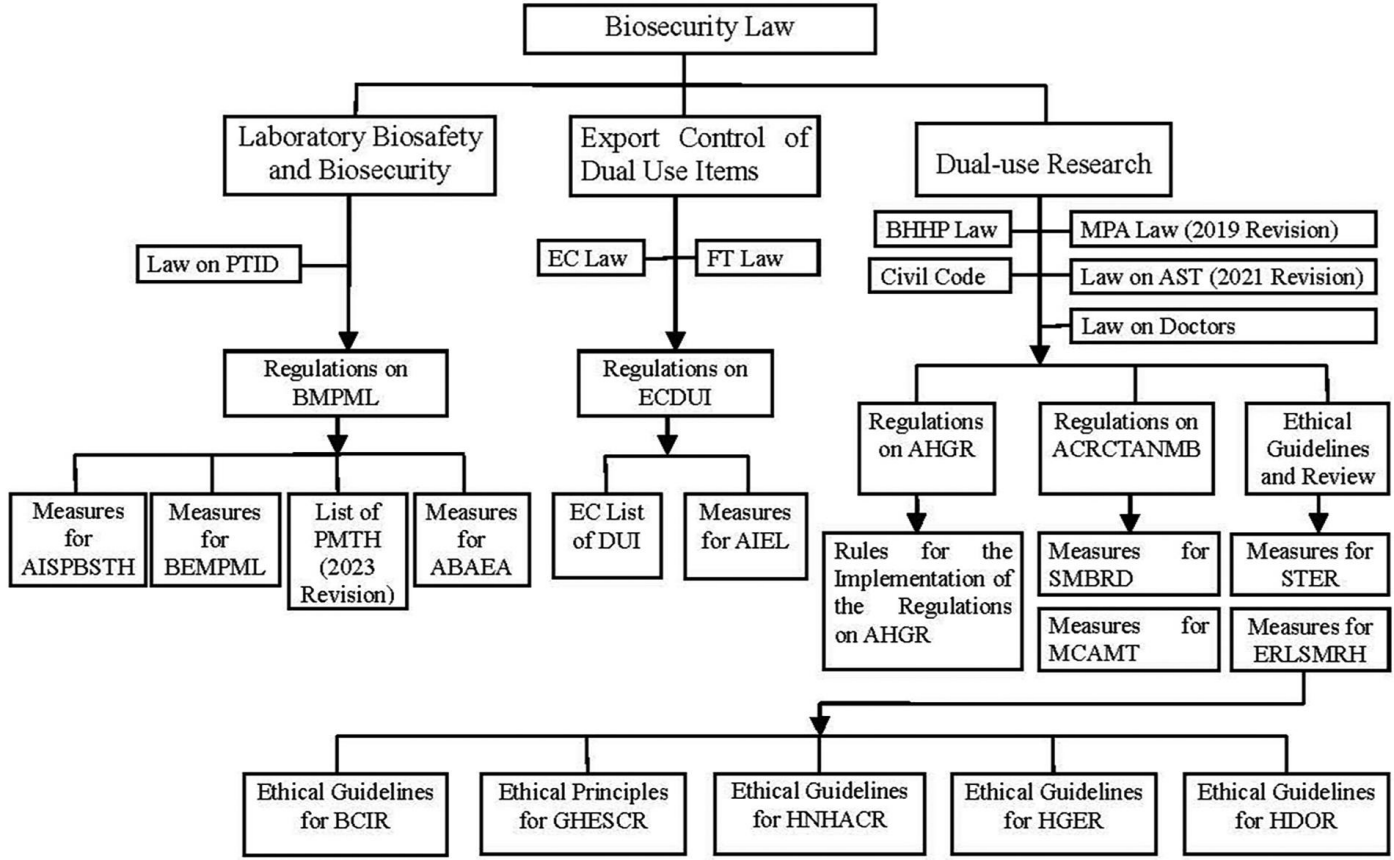
China's legal framework for medical biotech risk management. PTID, prevention and treatment of infectious diseases; EC, export control; FT, foreign trade; BHHP, basic healthcare and health promotion; MPA, medicinal product administration; AST, advancement of science and technology; BMPML, biosafety management of pathogenic microbe laboratories; ECDUI, export control of dual-use items; AHGR, administration of human genetic resources; ACRCTANMB, administration of clinical research on and clinical translational application of new medical biotechnologies; AISPBSTH, administration of institutions storing pathogenic bacterial (viral) strains transmitting among humans; BEMPML, biosafety environmental management of pathogenic microbe laboratories; PMTH, pathogenic microbes transmitting among humans; ABAEA, administration of the biosafety approval of experimental activities related to highly pathogenic microbes transmitting among humans; DUI, dual-use items; AIEL, administration on import and export license for dual-use items and technologies; SMBRD, safety management of biotech research and development; MCAMT, management of the clinical application of medical technologies; STER, scientific and technological ethical review; ERLSMRH, ethical review of life science and medical research involving humans; BCIR, brain computer interface research; GHESCR, guiding human embryonic stem cell research; HNHACR, human non-human animal chimera research; HGER, human genome editing research; HDOR, human derived organoids research.

China's current legal framework for medical biotech regulation is a three-tier system (Figure 1). At the top is the *Biosecurity Law* as the overarching law. It is complemented by various specific laws such as the *Law on the Prevention and Treatment of Infectious Diseases*, the *Basic Healthcare and Health Promotion Law*, the *Civil Code*, the *Export Control Law*, the *Amendment (XI) to the Criminal Law*, the *Law on the Advancement of Science and Technology*, etc. In the middle are the administrative regulations formulated by the State Council in accordance with relevant superior laws. At the bottom are implementing rules and measures issued by competent government ministries and agencies.

Underpinning the legal system is the regulatory philosophy of balancing innovation with security and emphasizing the equal importance of ethics and risk prevention. It regulates three categories of objects: persons, activities and items. The first category includes three kinds of persons. Legal persons (i.e., medical institutions) are required to establish a comprehensive academic, ethical, and risk management system. Researchers should have qualifications and comply with ethical norms. Subjects or patients should have the right to informed consent, free compensatory treatment, and privacy protection. The second category involves a full life cycle from preclinical and clinical research to clinical translational application in accordance with the risk classification of the activities concerned, with ethical review being a mandatory prerequisite and a continuous process for all human-related researches. Besides, a dynamic negative-list approach is adopted for both clinical researches and clinical translational applications, with the former subject to filing management and the latter subject to administrative approval. The third category covers pathogenic microorganisms, human genetic resources, and dual-use biological products and related technologies. The oversight of pathogenic microorganisms is conducted under a four-level risk grouping system with mandatory approval for the experiment, storage, and transportation of highly pathogenic microorganisms. The human genetic resources are managed under a central-provincial-local hierarchical filing and approval system, covering the entire process of collecting, preserving, utilizing, and outbound supply for international collaborative research. Dual-use biological products and related technologies are included in export control lists under a licensing system to prevent their proliferation and misuse.

### 3.2 Organizational structure

**Figure 2:**
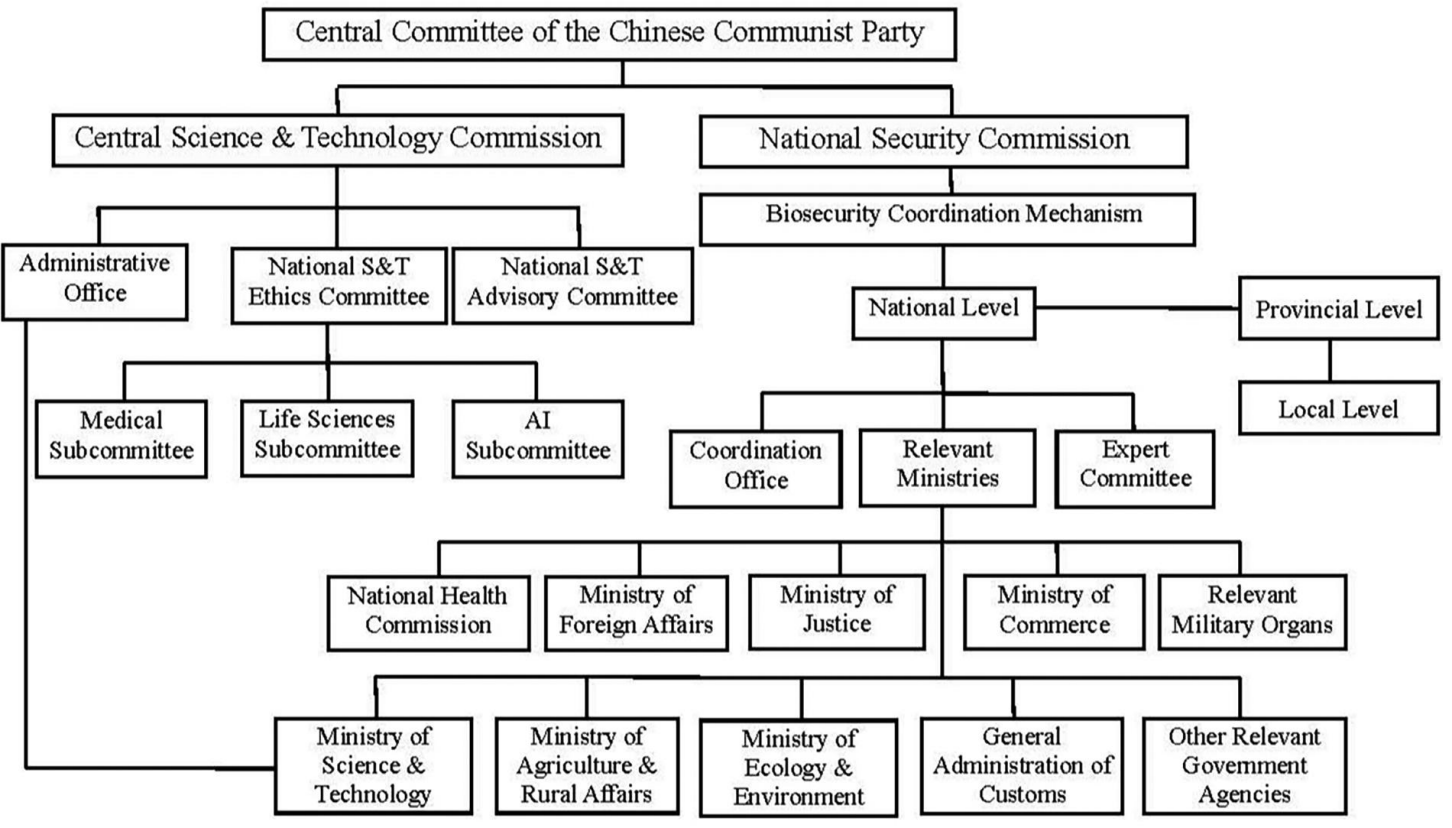
China's organizational structure for medical biotech risk management.

Two special organs under the Central Committee of the Chinese Communist Party are responsible for biosecurity supervision, i.e., the National Security Commission and the Central Science & Technology Commission (Figure 2). The National Security Commission is the highest decision-making body for national security affairs, including biosecurity. Underneath is the National Biosecurity Coordination Mechanism, which is led by the National Health Commission and composed of competent ministries in charge of health, agriculture, science, technology, diplomacy, and military affairs. Member agencies of this interagency mechanism are responsible for the biorisk management in their respective domains. For example, the National Health Commission is responsible for infectious disease prevention and control, and laboratory biosafety, among others; the Ministry of Science & Technology supervises human genetic resources, biotech research and development, high-level laboratory construction approval, etc. Under the National Biosecurity Coordination Mechanism, provincial and local coordination mechanisms are established.

The Central Science & Technology Commission is the highest decision-making body for national scientific and technological strategies, plans, and policies of significant importance. It has three subordinate agencies: the administrative office, the advisory committee, and the ethics committee. The Ministry of Science & Technology serves as its administrative office and its minister as head of the office. The National Science &Technology Ethics Committee was established in 2019 with subcommittees in three ethical sensitive areas: medical, life sciences and artificial intelligence. Its fundamental responsibilities include enhancing governance mechanisms, strengthening ethical oversight, refining relevant laws and ethical review rules, and standardizing various scientific research activities.

## 4 Major challenges

China's medical biotech regulatory regime has made remarkable progress in integration, systematization and legalization, forming a comprehensive framework led by the *Biosecurity Law* and linked with multi-level laws and regulations. Since the enactment of the *Biosecurity Law* in 2020, there have been no severe biosecurity incidents involving medical technologies, such as laboratory pathogen leaks or human gene editing. The most serious reported incident was a series of cases of smuggling pregnant women’s blood samples, which were adjudicated in 2025 under the criminal charge of smuggling human genetic resource materials pursuant to the *Amendment (XI) to the Criminal Law*. However, facing the reality of accelerated technological iteration, interdisciplinary integration, and the increasingly complex governance environment, the existing system still exposes structural deficiencies and insufficient adaptability. These challenges stem not only from the rapid evolution of biotechnology itself, but also reflect the deep-seated flaws in institutional design. Accurately identifying and deeply analyzing these challenges is a key prerequisite for promoting the regulatory regime to move from formal completeness to effective governance.

### 4.1 Convergence of AI and biotechnology

The increasing convergence of AI and medical science is intensifying the governance challenge of medical biotechnology (21). AI technologies for drug discovery could potentially be misused to identify toxic molecules (22). The same biological AI models can be trained both to design a benign viral vector to deliver gene therapy and to design a more pathogenic virus capable of evading vaccine-induced immunity (23). The dual-use nature of medical biotechnology and AI and their convergence require integrated, adaptive and resilient regulatory frameworks to address potential risks.

### 4.2 Ambiguity of regulatory boundaries

The *Regulations on the Administration of Clinical Research on and Clinical Translational Application of New Medical Biotechnologies* released in 2025 (*2025 Regulations on ACRCTANMB*) is the first administrative regulation that defines “new medical biotechnology”, namely, specialized medical methods and measures that utilize biological principles to act on human bodies at the cellular and molecular levels with a view to assessing health status, preventing or treating diseases, and promoting health, but which have not yet been clinically applied within China. But how to judge a technology is “new” or “has not been clinically applied” is not clear. For example, is the application of a technology in any (qualified) medical institution considered “clinically applied”? Or does it require widespread clinical adoption nationwide? On the other hand, a medical biotechnology is legally recognized as “clinically applied” when it is approved by the government for clinical translation and application, but the standards for the approval, as well as the rules for technical and ethical evaluation, remain to be clarified.

Besides, medical biotechnology is closely related to or overlapping with medical products and devices. For example, it is still unclear whether gene therapy products and stem cell preparations should be regulated as new medical biotechnologies or drugs. The ambiguity of the technical boundary among these concepts will cause regulatory overlaps or gaps.

### 4.3 Fragmentation and soft constraint of ethical regulations

China's current biosecurity legislation does attempt to strengthen ethical constraints on biomedical research activities. But two major issues need to be addressed. First, the ethical regulatory policies for science and technology are not systematically constructed with a clear purpose but are scattered across various laws in the form of sporadic provisions. While these provisions provide the legal base for the ethical oversight in specific fields, the relevant laws do not prioritize the biotech ethics in terms of value concepts, legislative objectives, or the formulation of fundamental principles. Second, the legal provisions primarily consist of such prohibitions or requirements as “should not violate ethical and moral standards” or “should comply with ethical principles”, but they neither specify the concrete connotations of such principles, norms or standards nor exercise direct mandatory intervention. On the other hand, ethical guidelines with which the laws require to comply are issued in the form of lower-level government agency policies or measures, while the National Science & Technology Ethics Committee, which formulates ethical guidance at the national level, is an academic and professional expert committee. Therefore, the existing ethical supervisory system is short of hard constraint and mainly relies on self-discipline.

### 4.4 Lack of transparency and public engagement

In a medical biotech regulatory system, the government, enterprises, the public, and experts are the most important stakeholders, each wielding distinct influence. Experts possess knowledge-based authority, shaping public and policymakers' perceptions. The government has the authority to formulate and implement policies, determining the scope and standards of technology application. The public holds the power of choice and pressure, influencing clinical applications and market potentials of the technology. Enterprises, as well as medical institutions, possess the capability to realize technological advancements and market profits, serving as the core drivers for clinical applications. In China, the public's influence is noticeably weak in both the policy formulation and implementation processes. For instance, decision-making processes, such as new regulation development, major clinical trial approvals, and ethical reviews, lack statutory public consultation or hearing procedures. Ethical review boards at various institutional levels are predominantly composed of biological and medical experts, with insufficient representation from the general public, patients, and social science scholars. Ethical review boards are required to submit annual reports to the government department, but merely for internal management and compliance purposes, rather than for public information disclosure. China has established a multi-tiered and periodic inspection system for high-level biological laboratories, requiring all registered laboratories to submit self-inspection results online via the “National Pathogen Microbiology Laboratory Management and Service Platform” to enable digitalized oversight. However, there are no legal provisions requiring publication of detailed inspection outcomes for each assessment.

## 5 Suggestions for further improvement

The further improvement of China's medical biotech regulatory regime needs to shift from passive- response governance to forward-looking adaptation. This not only requires institutional design to cope with the emerging dual-use risks brought about by the integration of AI and biotechnology, but also needs to correct the deficiencies of the existing system in terms of precision, constraint, and transparency.

### 5.1 Legislation on the convergence of AI and medical biotechnology

Currently, China lacks a unified basic law on AI, and the regulatory system is characterized by fragmentation and decentralization, just like that of medical biotechnology before the 2020s (24). The *2025 Regulations on ACRCTANMB* exclude AI-related medical biotechnologies if they neither act on human bodies at the cellular or molecular levels, nor are based on biological principles. The main guideline governing the convergence of AI and medical biotechnology is the *Opinions on Promoting and Regulating the Application and Development of “AI Plus Healthcare”* jointly issued in 2025 by the National Health Commission, the National Development & Reform Commission, the Ministry of Industry & Information Technology, the National Administration of Traditional Chinese Medicine, and the National Disease Control & Prevention Administration. It embodies the principles of balancing data security, algorithm compliance, and industry development, but is only a low-level agency guidance. Worldwide AI legislative experience shows that the institutional design among different countries is significantly different, but the central idea of AI governance is basically the same, i.e., proportionality based on different levels of risk (25). Likewise, China should develop a hierarchical AI regulatory regime with its own characteristics based on risk identification and classified supervision. The clinical research and application of new medical biotechnologies should be incorporated into the regime as one of the key AI application scenarios.

### 5.2 Clarification and streamlining of regulations

One of the key issues in China's medical biotech regulation is to distinguish “technologies” from “products” and “devices”. Basically, the *2025 Regulations on ACRCTANMB*, the *MPA Law (2019 Revision)*, and the *Regulations on the Supervision and Administration of Medical Devices (2024 Revision)* clarify their boundaries based on purpose-mechanism dimensions. Medical biotechnologies are methods and measures whose therapeutic mechanism is based on biological interventions at the cellular and molecular levels. Medical products are substances whose therapeutic mechanism is based on *in vivo* pharmacological, immunological, or metabolic effects. Medical devices are instruments, equipment, tools, *in vitro* diagnostic reagents and calibrators, materials, and other similar or related items directly or indirectly used for the human body whose therapeutic mechanism is based on *in vivo* or *in vitro* physical or mechanical processes or on the auxiliary roles in pharmacological, immunological, or metabolic prossesses. But “new” medical products or devices are definitely the outcome of “new” medical technologies. Accordingly, regulatory rules designed to distinguish their respective attributes should consider two additional dimensions: process and output. That is, a medical technology is an intangible output whose *service* process is inseparable from its recipients, i.e., patients or subjects, while a medical product or device is a tangible output whose *production* process is separable from its end-users. Take stem cell therapy as an example. When it is utilized by a medical institution as a clinical approach or scheme to supply personalized medical services to patients, it is a “technology”. But it is a “product” or “device” if employed by an enterprise as a means of production to produce standardized therapeutic drugs or tools.

### 5.3 Legalization of biotech ethical supervision

Globally, there are two main legislative modes for biotech ethics. One is to develop specialized biotech laws, which construct ethical review as a part of them, such as Germany's 1990 *Genetic Engineering Act* (26) and the UK's 1990 *Human Fertilization and Embryology Act* (27). The other is to establish a specialized *Bioethics Law* like France, which covers all ethical issues from birth to death, including biomedically assisted reproduction, genetic information acquisition, organ donation, and those arising from advances in biotechnology. China can learn from these legislative modes, or choose a third path. One alternative is to formulate a *Biotech Research and Application Law*, which would regulate the entire process of biotech research and application covering ethical review, risk assessment, classified supervision, information filing, and retrospective accountability.

For the ethical review and supervision, a four-tier collaborative framework should be established by the law. The first tier is the institutional ethical review board, conducting comprehensive review of all medical biotechnology projects proposed by the institution. The second tier is the local administrative authority, adding additional oversight reviews for biotech applications that may violate legal prohibitions or pose significant ethical challenges. The third tier is the National Science & Technology Ethics Committee, responsible for drafting and adjusting the list of technologies requiring additional oversight reviews and providing advisory opinions to local regulatory agencies. At the top is the general public, offering feedback and oversight on both misconduct during the review process and potentially illegal applications of biotechnology.

### 5.4 Institutionalization of regulatory transparency and public engagement

Within the current medical biotech regulatory framework, China should strengthen the procedural, standardized, and institutionalized design of information disclosure and public engagement. First, for existing policies and regulations, national authorities overseeing biosafety and biosecurity of laboratories, import and export of dual-use items, and ethical review of medical researches and applications should regularly publish reports communicating operational metrics to the public. Second, during policy formulation and revision, procedures such as public consultations and hearings should be introduced. The hearing procedure should cover the entire process of announcement release, representative selection, presentation and debate, and result disclosure. The selection of public representatives should specify the methods, qualifications, quotas, and procedures, among others. Third, the *Interim Measures for Scientific and Technological Ethical Review* and the *2023 Measures for ERLSMRH* should be revised to include public representatives and social scholars in the institutional ethical review board with a view to diversifying its member composition. For example, the U.S. National Institutes of Health policy requires such boards to include non-institutional members who represent the interests of the local community (9). Meanwhile, the review board should be required to release regular reports to enable the public to access information related to the review activities and make recommendations. Fourth, the Ministry of Science & Technology as the executive body of the Central Science & Technology Commission, and the National Health Commission as the leading agency of the National Biosecurity Coordination Mechanism, should coordinate the construction of an online oversight platform for emerging medical biotechnologies. Just like the U.S. Unified Website for Biotechnology Regulation, the goal of the platform is to ensure public confidence in the regulatory system and improve transparency, predictability, coordination, and efficiency of the biotech regulatory system. Specifically, it should regularly disclose the information on approval filing, adverse events, ethical reviews, etc., and achieve real-time data sharing across departments and agencies. Moreover, in the course of policy formulation, transparent forums should be created at the platform to address scientific, safety, and ethical issues, thereby incorporating public insights and addressing social concerns.

## 6 Conclusion

China's medical biotech regulatory regime has followed the evolving path of exploration, development, and maturity since the 1980s. The regulatory focus has expanded from laboratory biosafety and biosecurity to dual-use item export control, human genetic resource management, and risk oversight of dual-use research and application. The regulatory philosophy has shifted from crisis-driven passive response to proactive risk prevention.

Currently, the regime is supported by multi-tiered laws and regulations, and executed by specialized coordination mechanisms. The legal framework adopts a three-tier structure of “law—regulation—rule”, which covers three categories of objects: persons, activities, and items and implements a lifecycle management based on the risk classification. In terms of organizational structure, the National Biosecurity Coordination Mechanism and the Central Science & Technology Commission have been established. They oversee the responsibilities of government departments at all levels for infectious disease prevention and control, laboratory safety and security, human genetic resource management, and science and technology ethics. Additionally, the National Science & Technology Ethics Committee and its subcommittees have been set up to strengthen ethical review and guidance in sensitive areas like life sciences and medical science.

However, the future development of China's medical biotech regulatory regime will still face several challenges. First, the convergence of AI and biotechnology intensifies the risks of technological misuse and governance complexity. Second, the ambiguous definition of “new medical biotechnologies” creates unclear boundaries with medical products and devices, leading to regulatory overlaps or gaps. Third, ethical review provisions are scattered across various laws, mostly consisting of general principles, while detailed guidelines are often issued by lower-level government agencies or expert committees, lacking legal enforceability and systematic coherence. Fourth, the transparency of policy formulation and implementation processes is insufficient, with weak public participation mechanisms. The absence of public and community representation in ethical review boards of medical institutions, along with the lack of mandatory disclosure of oversight outcomes, further exacerbates these issues.

To address those challenges, four countermeasures are recommended. First, a specialized law on the convergence of AI and biotechnologies should be formulated based on the risk identification and classified supervision of AI application scenarios. Second, technical boundaries among “new” medical biotechnologies, products, and devices should be clarified based on multiple dimensions of “purpose—mechanism—process—output” to avoid regulatory overlaps or gaps. Third, the legalization of biotech ethical supervision should be accelerated by formulating a specialized law to clarify ethical review, risk assessment, and accountability mechanisms. Fourth, regulatory transparency and public engagement should be enhanced to promote social supervision and consensus building by means of introducing hearing and consultation procedures into the policy-making process, optimizing the composition of institutional ethical review boards, and establishing information disclosure platforms.
